# Progressive supranuclear palsy can mimic idiopathic normal pressure hydrocephalus: A case series

**DOI:** 10.1093/jnen/nlad090

**Published:** 2023-11-07

**Authors:** Miki Kawazoe, Shunsuke Koga, Dennis W Dickson

**Affiliations:** Department of Neuroscience, Mayo Clinic, Jacksonville, Florida, USA; Department of Neuroscience, Mayo Clinic, Jacksonville, Florida, USA; Department of Neuroscience, Mayo Clinic, Jacksonville, Florida, USA

To the Editor:

Idiopathic normal pressure hydrocephalus (iNPH) is characterized by a triad of gait disturbance, cognitive impairment, and urinary incontinence. It is typically accompanied by enlarged ventricles and disproportionately enlarged subarachnoid space on neuroimaging (1). The clinical features of iNPH can mimic neurodegenerative disorders, such as progressive supranuclear palsy (PSP), Lewy body disease, or Alzheimer disease (2–5). Although curative treatments for these diseases are lacking, ventriculoperitoneal shunt (VPS) surgery has been established as a treatment for iNPH (6). Therefore, it is important to differentiate iNPH from neurodegenerative disorders because VPS may benefit patients with iNPH, but not patients with other neurodegenerative disorders.

We identified 2 patients with iNPH who at autopsy had neuropathologic findings of PSP. The first patient was a 70-year-old man with a 6-year history of Parkinsonism with progressive gait difficulties. He also had poor balance, short-term memory decline, urinary urgency, dysarthria, hypophonia, and depression. His past medical history was notable for hyperlipidemia, and his family history was significant for Parkinsonism in his mother. On neurological examination 1 year before he died, he had normal language, memory, and fund of knowledge, but his cognitive responses were slow. His cranial nerve exam was unremarkable, including exams of extraocular motility, but he had masked facies. His motor function and tendon deep tendon reflexes were normal. He had a slow, shuffling gait, decreased stride length, and no arm swing. An MRI of the brain showed mild generalized cerebral atrophy with ventriculomegaly that was disproportionate to sulcal dilatation. A high-volume lumbar puncture showed dramatic, temporary improvement in his symptoms, leading to the diagnosis of iNPH. He underwent VPS placement 2 years before he died; however, improvement in his symptoms lasted only for several weeks.

**Figure 1:**
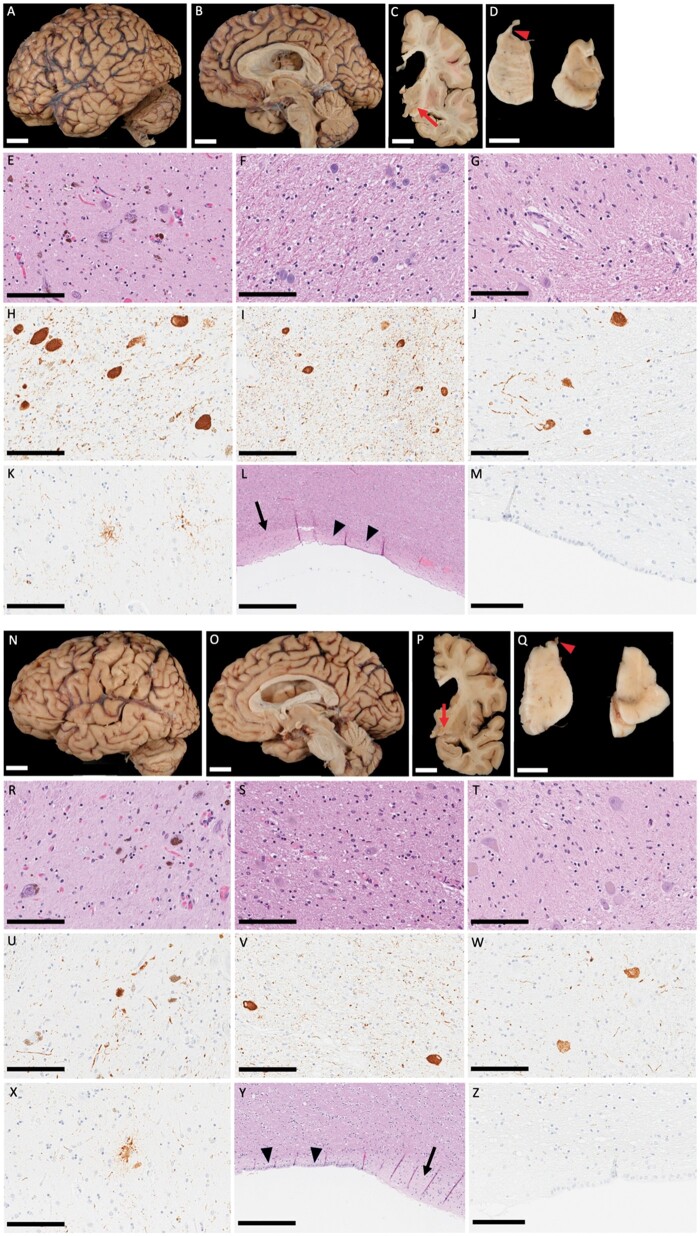
Macroscopic and histopathologic images (Case 1: **A–M**, Case 2: **N–Z**). *Case 1:* Macroscopically, the brain shows no focal atrophy (**A**). Marked midbrain atrophy (**B**) with enlargement of the lateral ventricle is shown (**B**, **C**) and the subthalamic nucleus has marked atrophy and red-brown discoloration (arrow) (**C**). The substantia nigra has decreased pigmentation and the superior cerebellar peduncle has severe atrophy (arrowhead) with enlargement of the fourth ventricle (**D**). Histopathologically, the substantia nigra (**E**), subthalamic nucleus (**F**), and dentate nucleus (**G**) show mild to severe neuronal loss. Immunohistochemistry for phospho-tau (CP13) shows pretangles in the substantia nigra (**H**), subthalamic nucleus (**I**), and dentate nucleus (**J**) and shows coiled bodies in the subthalamic nucleus (**I**). Tufted astrocytes are seen in globus pallidus (**K**). The ependymal lining of the lateral ventricles (arrowhead) has severe discontinuities and mild gliosis (arrow) (**L**); however, no significant tau-positive deposit is seen in the ependymal lining (**M**). *Case 2:* Macroscopically, mild cortical atrophy over dorsolateral frontal lobe is found (**N**). Midbrain atrophy with marked enlargement of frontal horn of the lateral ventricle and the dilatation of the aqueduct of Sylvius are shown (**O**, **P**). The subthalamic nucleus has atrophy and discoloration (arrows) (**P**). The substantia nigra has decreased pigmentation, especially lateral parts and the superior cerebellar peduncle has mild atrophy (arrowhead) (**Q**). Histopathologically, the substantia nigra (**R**) and subthalamic nucleus (**S**) show moderate to severe neuronal loss and gliosis, while the dentate nucleus is relatively preserved (**T**). Immunohistochemistry for phospho-tau (CP13) shows pretangles in the substantia nigra (**U**), subthalamic nucleus (**V**), and dentate nucleus (**W**). Tufted astrocytes are seen in globus pallidus (**X**). The ependymal lining of the lateral ventricles (arrowhead) has severe discontinuities and mild gliosis (arrow) (**Y**); however, no significant tau-positive deposit is seen in the ependymal lining (**Z**) (tau immunohistochemistry: E, F, G, L, R, S, T, Y: H&E: H, I, J, K, M, U, V, W, X, Z). Scale bars: A–D, N–Q = 2 cm; E–K, M, R–X, Z = 200 μm; L, Y = 300 μm.

At autopsy his brain weighed 1340 g and macroscopic examination did not reveal any atrophy (Fig. 1A) except for midbrain atrophy typical of PSP (Fig. 1B–D). The subthalamic nucleus had marked atrophy and discoloration (Fig. 1C). The neocortex had no significant neuronal loss or gliosis, except in the vicinity of the VPS track. The substantia nigra had marked neuronal loss (Fig. 1E). The cerebellar dentate nucleus had neuronal loss (Fig. 1G) and gliosis, with moderate to marked grumose degeneration. Immunohistochemistry for phospho-tau confirmed the findings of PSP with pretangles, globose tangles, tufted astrocytes, and coiled bodies in the substantia nigra, subthalamic nucleus, globus pallidus, the motor and premotor cortices, ventral thalamus, corpus striatum, the inferior olivary nucleus, pons, and cerebellar dentate nucleus (Fig. 1H–K) (Table). The pathologic findings were those of PSP. Additionally, there were several discontinuities in the ependymal lining of the lateral ventricles and mild gliosis (Fig. 1L, M), which has been reported in autopsies of iNPH (7).

**Table.** The distribution and severity of tau pathology

	NFT and pre-NFT	Coiled bodies	Tufted astrocytes	Tau-positive threads
	Case 1 − Case 2			
Temporal cortex	1+ **−** 1+	1+ **−** 1+	0 **−** 1+	0 **−** 0
Superior frontal gyrus	2+ **−** 2+	2+ **−** 2+	1+ **−** 2+	0 **−** 1+
Motor cortex	2+ **−** 2+	1+ **−** 2+	1+ **−** 1+	1+ **−** 2+
Caudate nucleus and putamen	2+ **−** 2+	2+ **−** 1+	2+ **−** 2+	1+ **−** 1+
Globus pallidus	3+ **−** 2+	2+ **−** 0	0 **−** 0	3+ **−** 0
Basal nucleus	3+ **−** 2+	1+ **−** 0	0 **−** 0	3+ **−** 2+
Hypothalamus	3+ **−** 3+	1+ **−** 0	0 **−** 0	2+ **−** 1+
Ventral thalamus	3+ **−** 2+	3+ **−** 2+	1+ **−** 0	3+ **−** 3+
Subthalamic nucleus	3+ **−** 3+	2+ **−** 2+	0 **−** 0	3+ **−** 3+
Thalamic fasciculus	0 **−** 0	3+ **−** 3+	0 **−** 0	3+ **−** 3+
Red nucleus	3+ **−** 2+	3+ **−** 2+	1+ **−** 1+	3+ **−** 3+
Substantia nigra	3+ **−** 4+	1+ **−** 1+	0 **−** 0	3+ **−** 2+
Oculomotor complex	3+ **−** 3+	0 **−** 1+	0 **−** 1+	2+ **−** 3+
Midbrain tectum	2+ **−** 3+	3+ **−** 2+	2+ **−** 2+	3+ **−** 3+
Locus ceruleus	3+ **−** 3+	0 **−** 0	0 **−** 0	3+ **−** 3+
Pontine tegmentum	3+ **−** 3+	3+ **−** 2+	0 **−** 0	3+ **−** 3+
Pontine base	3+ **−** 3+	1+ **−** 1+	0 **−** 0	2+ **−** 2+
Medullary tegmentum	3+ **−** 3+	3+ **−** 0	0 **−** 0	3+ **−** 2+
Inferior olivary nucleus	3+ **−** 3+	2+ **−** 1+	1+ **−** 0	4+ **−** 3+
Dentate nucleus	3+ **−** 3+	0 **−** 1+	0 **−** 0	2+ **−** 2+
Cerebellar white matter	0 **−** 0	2+ **−** 2+	0 **−** 0	1+ **−** 1+

The severity of each tau lesion is shown on a 5-point scale: 0 = none; 1+ = mild; 2+ = moderate; 3+ = severe; 4+ = very severe.

The second patient was a 79-year-old woman with a 5-year history of gait difficulties, poor balance, and multiple falls. Her past medical history was notable for anticardiolipin antibody syndrome and hypertension. Brain imaging showed cortical atrophy and ventriculomegaly. She had VPS 5 years before she died of presumed iNPH, which was associated with improvement in her balance and gait immediately afterward, but it lasted only a month. On neurological examination after the surgery, she had spontaneous laughter, decreased memory, decreased pursuit eye movements in the vertical plane, and square wave jerks on sustained gaze. She fell frequently, usually backward. She developed a hoarse voice, and she became impulsive. Her motor exam was notable for mild increased tone in the right arm, mild resting tremor in the head, and postural tremors in bilateral hands. She had no hyperreflexia and ataxia. She had a positive applause sign. A subsequent MRI showed midbrain atrophy and a hummingbird sign consistent with PSP.

At autopsy, her brain weighed 1120 g and there was mild dorsolateral frontal and posterior cortical atrophy (Fig. 1N). There was also midbrain atrophy (Fig. 1O). Sequential coronal sections revealed marked enlargement of the frontal horn and mild enlargement of the temporal horn of the lateral ventricle, as well as dilatation of the aqueduct of Sylvius (Fig. 1P). The substantia nigra had decreased pigmentation, and there was atrophy of superior cerebellar peduncle (red arrowhead) (Fig. 1Q). On hematoxylin and eosin-stained sections, there was a surgical defect (VPS site) with tissue loss and gliosis in the frontal cortex. Moderate-to-severe neuronal loss and gliosis were observed in the globus pallidus, substantia nigra, midbrain tectum, and subthalamic nucleus (Fig. 1R, S), while the cerebellar dentate nucleus was relatively preserved (Fig. 1T). Phosphorylated-tau immunohistochemistry showed pathology consistent with PSP, but tau pathology was milder than in most cases of PSP (Fig. 1U–X) (Table), while there was marked pallido-nigro-luysial degeneration (8). In addition to PSP, there were discontinuities of the ependymal lining of the lateral ventricles and mild subependymal gliosis (Fig. 1Y, Z) (7).

Differentiating iNPH from other neurodegenerative disorders can be challenging due to overlapping clinical and imaging features, particularly when neuroimaging reveals enlarged ventricles. Whether iNPH mimics or coexists with other neurodegenerative diseases remains controversial (3). One study reported 4 patients with possible iNPH and autopsy findings of PSP or Parkinson disease with only temporary improvement in gait and cognition for several weeks after VPS (9). In contrast, Saitoh et al (7) reported a patient with NPH and pathologically confirmed corticobasal degeneration who had more than 1 year of benefit from VPS. Neither of our patients had sustained benefit from VPS. The temporary effectiveness of VPS may be attributed to the potential comorbidity of iNPH (10).

Despite the efficacy of VPS surgery in treating iNPH, it remains unclear whether it has beneficial effects in patients with iNPH and concurrent neurodegenerative disorders (3). The response to VPS shunt may vary depending on whether the primary pathology is a neurodegenerative disorder or iNPH. Therefore, careful clinical assessment is necessary to avoid overlooking patients who may benefit from VPS.
